# Genotype-specific effects of *Mecp2* loss-of-function on morphology of Layer V pyramidal neurons in heterozygous female Rett syndrome model mice

**DOI:** 10.3389/fncel.2015.00145

**Published:** 2015-04-20

**Authors:** Leslie Rietveld, David P. Stuss, David McPhee, Kerry R. Delaney

**Affiliations:** Department of Biology, University of VictoriaVictoria, BC, Canada

**Keywords:** pyramidal neuron, Sholl analysis, dendrites, Rett syndrome, female mouse, X-linked genetic disease, MeCP2

## Abstract

Rett syndrome (RTT) is a progressive neurological disorder primarily caused by mutations in the X-linked gene methyl-CpG-binding protein 2 (*MECP2*). The heterozygous female brain consists of mosaic of neurons containing both wild-type MeCP2 (MeCP2+) and mutant MeCP2 (MeCP2-). Three-dimensional morphological analysis was performed on individually genotyped layer V pyramidal neurons in the primary motor cortex of heterozygous (*Mecp2^+/-^*) and wild-type (*Mecp2^+/+^*) female mice ( > 6 mo.) from the *Mecp2^tm1.1Jae^* line. Comparing basal dendrite morphology, soma and nuclear size of MeCP2+ to MeCP2- neurons reveals a significant cell autonomous, genotype specific effect of *Mecp2.* MeCP2- neurons have 15% less total basal dendritic length, predominantly in the region 70–130 μm from the cell body and on average three fewer branch points, specifically loss in the second and third branch orders. Soma and nuclear areas of neurons of mice were analyzed across a range of ages (5–21 mo.) and X-chromosome inactivation (XCI) ratios (12–56%). On average, MeCP2- somata and nuclei were 15 and 13% smaller than MeCP2+ neurons respectively. In most respects branching morphology of neurons in wild-type brains (MeCP2 WT) was not distinguishable from MeCP2+ but somata and nuclei of MeCP2 WT neurons were larger than those of MeCP2+ neurons. These data reveal cell autonomous effects of *Mecp2* mutation on dendritic morphology, but also suggest non-cell autonomous effects with respect to cell size. MeCP2+ and MeCP2- neuron sizes were not correlated with age, but were correlated with XCI ratio. Unexpectedly the MeCP2- neurons were smallest in brains where the XCI ratio was highly skewed toward MeCP2+, i.e., wild-type. This raises the possibility of cell non-autonomous effects that act through mechanisms other than globally secreted factors; perhaps competition for synaptic connections influences cell size and morphology in the genotypically mosaic brain of RTT model mice.

## Introduction

Rett Syndrome (RTT) is a postnatal neurological disorder that was first characterized by Rett (1966). It affects approximately one in 10,000 live female births (Laurvick et al., 2006). Typical RTT patients are diagnosed after a period of normal development (6–18 months of age) followed by a period of regression marked by four main criteria: partial or complete loss of purposeful hand movements, development of gait abnormalities, loss of verbal language skills and the presence of stereotypic hand movements (Neul et al., 2010). The majority of females with RTT are heterozygous for a mutation in the X-linked gene Methyl-CpG-binding Protein 2 (*MECP2*; Amir et al., 1999). Normally, *MECP2* expression levels are low prenatally, but increase after birth during the final stages of neurogenesis (Balmer et al., 2003; Kishi and Macklis, 2004; Skene et al., 2010), supporting the observation that MeCP2 contributes to neuronal dendritic maturation and synaptogenesis (Armstrong et al., 1995; Kishi and Macklis, 2004; Fukuda et al., 2005). Although over 1000 mutations have been characterized along the entire length of the gene, including nonsense, missense, frameshift, and large truncation mutations (Amir and Zoghbi, 2000; Weaving et al., 2005; Philippe et al., 2006; Cuddapah et al., 2014), 65% of RTT cases are caused by eight common missense mutations in the region that encodes the methyl-CpG binding domain (MDB) of MeCP2 (Miltenberger-Miltenyi and Laccone, 2003).

The protein encoded by the *MECP2* gene has five key domains including a highly conserved MDB characteristic of its protein family, a transcriptional repressor domain, which interacts with histone deacetylases 1 and 2 to promote chromatin condensation, and a C-terminal domain, which contributes to DNA-binding (Chandler et al., 1999). Phenotype/genotype studies have found that early N-terminal truncation mutations that affect the MBD are correlated with a more severe phenotype than late C-terminal truncation mutations (Zappella et al., 2001; Charman et al., 2005; Cuddapah et al., 2014). The *Mecp2^tm1.1Jae^* mice used in this study have exon 3 deleted, which comprises most of the MBD. The resulting translated mutant protein may be partly functional (Stuss et al., 2013), leading to a milder phenotype in these animals than has been observed in total knockouts (Belichenko et al., 2009), but still resulting in severe neuropathic symptoms similar to the human condition (Chen et al., 2001).

MeCP2 is found in many tissue types, but it is most abundant in the brain, with expression levels in neuronal nuclei ten times higher than in glia (Skene et al., 2010). *MECP2* is located on the X-chromosome and therefore it is affected by X-chromosome inactivation (XCI; Adler et al., 1995; Ross et al., 2005). XCI occurs in early embryogenesis (gastrulation in humans), randomly inactivating either the maternal or paternal X-chromosome in each cell and passing this status on to all future progeny (Bermejo-Alvarez et al., 2012). XCI therefore results in two populations of neurons in RTT females: those expressing wild-type MeCP2 (MeCP2+), and those that lack fully functional MeCP2 (MeCP2-) Although the majority of human patients with typical RTT have nearly balanced XCI ratios (Shahbazian et al., 2002), the rate of skewed XCI ratios is nonetheless higher in patients with X-linked disorders such as RTT than in the general population. When skewing occurs it usually favors wild-type over mutant cells (Belmont, 1996; Puck and Willard, 1998; Brown and Robinson, 2000) and XCI ratios follow a similar pattern in mice (Young and Zoghbi, 2004).

Rett syndrome patients deficient in MeCP2 have reduced gray matter volume in the frontal and temporal lobes, caudate nucleus, thalamus, midbrain, and cerebellum (Reiss et al., 1993; Subramaniam et al., 1997). Volume reductions in the frontal and temporal lobes have been found to be predictive of phenotype severity in RTT (Carter et al., 2008). Neurodegeneration has not been observed in forebrain (Armstrong, 1995), and the symptoms of RTT in transgenic mice can be reversed even in adulthood (Luikenhuis et al., 2004; Guy et al., 2007, 2010) suggesting that RTT is caused by a defect in neurological function rather than by neuronal damage (McGraw et al., 2011). Morphological studies performed on post-mortem tissue from both RTT patients and mouse models have revealed that the reduction in brain volume is accompanied by increased neuron density associated with reductions in neuronal cell body size (Bauman et al., 1995; Chen et al., 2001; Goffin et al., 2011; Wang et al., 2013). It has also been found that RTT patients have reduced dendritic arborization and spine density of Layer II/III and V/VI pyramidal cells in the frontal and temporal areas, including the motor cortex (Belichenko et al., 1994, 1997). Similar reductions in spine density have been reported for hippocampal CA1 neurons of RTT patients (Chapleau et al., 2009).

Most animal studies have used *Mecp2* mutant male mice (*Mecp2^-/y^*), since their juvenile symptom onset and rapid progression mirrors RTT patients and shortens the time required for experimental studies. As well, since *Mecp2^-/y^* mice express mutant MeCP2 in all cells, variations in XCI ratio between individuals are not a potential complication. However, these studies may not address distinct effects that occur in the mosaic brain environment of heterozygous females. *Mecp2^+/-^* mice display the same RTT-like neuropathological and motor abnormalities as *Mecp2^-/y^* mice, but these symptoms are normally only begin to become apparent after 4–6 months of age, compared to 3–5 weeks for male mice, with some female mice even living a normal lifespan (Chen et al., 2001; Guy et al., 2001, 2007; Bissonnette and Knopp, 2006; Bissonnette et al., 2007; Abdala et al., 2010). However, progressive motor deficits, reduced anxiety, apnea, and weight gain have been described in *Mecp2^+/-^* mice as young as 3–4 weeks old (Santos et al., 2007; Samaco et al., 2009, 2012), which are largely pre-symptomatic for the more readily observed motor abnormalities.

The mosaic brain environment of the female *Mecp2^+/-^* mouse prompts the following question: are the cellular phenotypes of MeCP2+ and MeCP2- neurons determined by their individual genotype (cell autonomy) or by the surrounding environment, including neural and non-neuronal cells (non-cell-autonomy), or both?

Our laboratory recently investigated the morphology of pyramidal neurons in the primary motor cortex of *Mecp2^tm1.1Jae^* male mice using the YFP-H transgenic mouse line (Feng et al., 2000) to visualize morphology. We found that *Mecp2^-/y^* basal and apical dendritic arbors are reduced in length, and that *Mecp2^-/y^* spine density is selectively reduced on the apical tuft and on oblique apical dendrites (Stuss et al., 2012). The aim of the current study is to investigate how a mutation in *Mecp2* affects the morphology of both neuronal genotypes in a female *Mecp2^+/-^* brain. We examined the morphology of basal dendrites, as well as the sizes of soma and nuclei in Layer V pyramidal neurons of the primary motor cortex, using single-cell microinjection of Alexa Fluor 594 with DAPI and Neurotrace^TM^ counterstains. Neuronal genotypes were determined by immunohistochemistry. For this study MeCP2- is used to indicate neurons within the brain of *Mecp2*^+/^*^-^* animals that express mutant MeCP2, MeCP2+ refers to neurons expressing wild-type MeCP2 and MeCP2 WT denotes neurons in wild-type female brain. Reduced branching was observed primarily within 30–70 μm of the soma in MeCP2- neurons. In most respects dendritic structures of MeCP2+ neurons were not distinguishable from MeCP2 WT neurons, indicating predominantly cell-autonomous effects of the mutation on dendrite branching. Within-animal comparisons showed that soma and nuclear sizes of MeCP2- neurons were reduced compared to MeCP2+ neurons, consistent with cell-autonomous effects. Between-animal comparisons revealed that MeCP2 WT somata were larger than MeCP2+, suggesting an additional non-cell-autonomous effect. Intriguingly, the ratio of MeCP2- to MeCP2+ soma and nuclear size was negatively correlated with XCI. The difference in size was greatest when the proportion of MeCP2- neurons was low (highly skewed XCI), which may indicate an additional negative environmental effect for MeCP2- neurons when they are in the minority or a reduced ability of MeCP2- neurons to benefit from a predominantly wild-type brain environment.

## Materials and Methods

### Experimental Animals

Heterozygous *Mecp2^+/-^* female mice (*Mecp2^tm1.1Jae^*/Mmcd; MMRRC, UC Davis; Chen et al., 2001) were maintained on a C57BL/6 background and their wild-type littermates (*Mecp2*^+/+^) were used as controls for soma and nuclear size analysis. *Mecp2^+/-^* females were crossed with homozygous male YFP-H mice [B6.Cg-Tg(Thy1-YFPH)2Jrs/J; Feng et al., 2000], which were also maintained on a C57BL/6 background, to generate female offspring heterozygous for both *Mecp2* and YFP-H. These offspring (*Mecp2^+/-^*/*YFP*^+/^*^-^*) and their wild-type littermates (*Mecp2*^+/+^/*YFP*^+/-^) were used for single-cell microinjection experiments. YFP expression observed with blue excitation confirmed the location of Layer 5 neurons for injection with Alexa 594. All animals were housed in the University of Victoria Animal Care Unit in compliance with the guidelines established by the Canadian Council on Animal Care and Use with approval of protocols by the University Animal Care Committee. Animals were kept at 21 ± 2°C under a 12 h light/dark cycle with limited environmental enrichment, and were fed standard laboratory diet and water *ad libitum. Mecp2* status was determined by genotyping (Stuss et al., 2012).

Prior to tissue extraction a phenotypic score was calculated by summing the severity scores for 6 characteristics (in parentheses) in **Table 1**. Mice were then anesthetized and transcardially perfused with 10 ml of room temperature 0.1 M phosphate buffered saline (PBS) pH 7.4 for exsanguination (10 ml/min).

**Table 1:** Phenotype severity scale for the Jaenisch Rett syndrome mouse model (deletion of exon 3 of *Mecp2*).

Parameter	Severity			
**Tremors:** observed while standing on the palm	No tremor **(0)**	Intermittent mild tremor **(1)**	Continuous tremor or intermittent violent tremor **(2)**
**Hind-clasping:** observed when suspended by holding the base of the tail	Legs splay outwards **(0)**	Legs are drawn inwards slightly **(1)**	Legs are drawn inwards but do not touch **(2)**	Legs are drawn inward and touch each other and the body tightly **(3)**
**Dishevelled fur**	Clean and shiny coat **(0)**	Coat dull/ungroomed **(1)**	Piloerection, dull coat/ungroomed **(2)**
**Activity level:** observed when placed on the bench	WT activity level **(0)**	Slower pace than WT but still active **(1)**	Still for periods, but can move slowly **(2)**	No spontaneous movements **(3)**
**Breathing problems**	Normal breathing **(0)**	Short periods of rapid breathing or apnea **(1)**	Irregular breathing, gasping or panting **(2)**
**Hunched**	WT rounded posture **(0)**	Slightly hunched **(1)**	Hunched posture **(2)**

*Adapted from (Guy et al., 2007).*

For mice prepared for single-cell microinjections, PBS perfusion was followed by 30 ml of 4% paraformaldehyde (4% PFA) in 0.1 M phosphate buffer, pH 6.5, at a rate of 5 ml/min. After removal from the cranium, the brain was post-fixed in 4% PFA, pH 6.5, for 10 min at RT. Following this, the brain was removed and placed in a weigh boat for up to 15 min before slicing.

For mice prepared for soma and nuclear size analysis (6–21 months), the PBS was followed by 10 ml of 4% PFA pH 6.5, (5 ml/min) and 20 ml of 0.1 M borate buffered 4% PFA, pH 11 at a rate of 2 ml/min. After craniotomy, the brain was submerged in 4% PFA, pH 11, for 2 h at RT. The brain was then immersed in 30% sucrose in PBS and 0.01% sodium azide at 4°C for ∼48 h.

Brains were embedded in agar and sliced coronally on a Vibratome Series 1000. The primary motor cortex was identified by the first appearance of the corpus callosum anteriorly and by the joining of the anterior commissure posteriorly (∼2 mm). Brains prepared for single cell microinjections were sliced into ∼10 200 μm-thick sections and then placed in 24-well plates filled with PBS and 0.01% sodium azide for storage at 4°C. Brains prepared for soma and nuclear size analysis were sliced into 50 μm-thick sections and placed in 24-well plates filled with 30% sucrose in PBS and 0.01% sodium azide for storage at 4°C prior to mounting.

**FIGURE 1:**
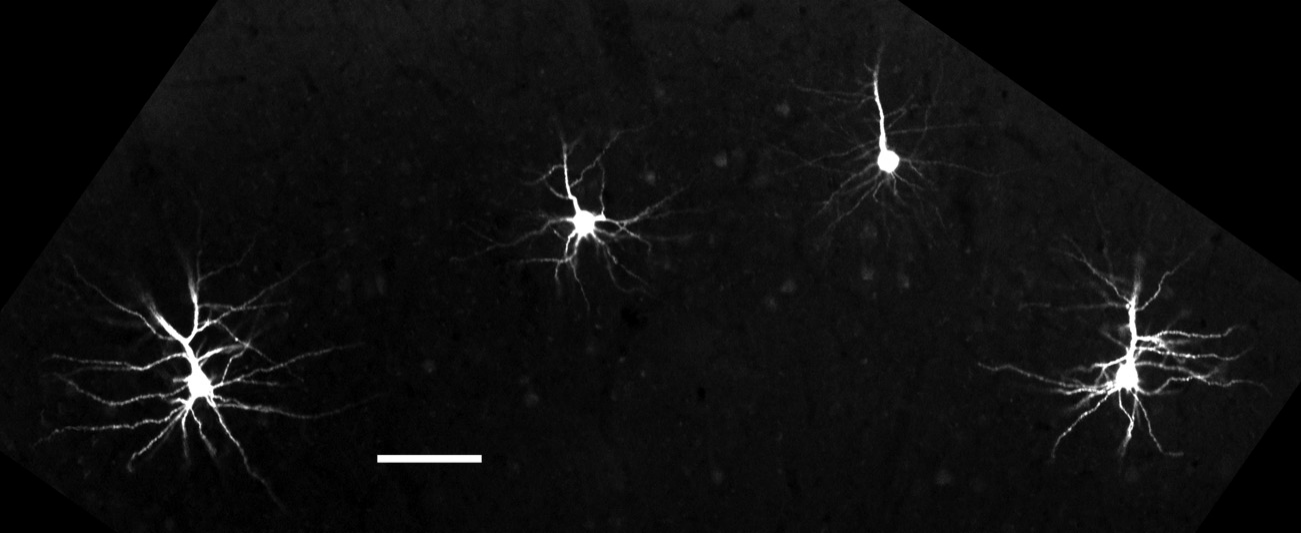
**Single cells microinjected with Alexa Fluor 594 dye.** Neurons selected for filling were distributed across brain slices to maximize the total number of visible cells per slice while preventing overlap of dendrites. Scale bar = 100 μm.

For microinjection, the slices were placed in a PBS bath. The cells were visualized using a custom-made epifluorescence microscope equipped with an Olympus UPlanFl 4X/0.13 NA lens and an Olympus LUMPlanFl 40X/0.8 NA water immersion lens. Borosilicate glass pipettes (O.D. 1.5 mm ID 0.86 mm; Sutter Instruments) were prepared with a resistance of 5–10 MΩ when filled with 120 mM KCl (∼0.5 μm tip opening) using a micropipette puller (Sutter Instruments P-87). Pipettes were backfilled by dipping in a solution of 3 mM Alexa Fluor 594 hydrazide dye (Invitrogen). Dye fluorescence was excited using a bandpass filter (HQ550/100, Chroma Optical) and visualized through a 624/40 filter (Semrock Brightline^TM^) using an analog CCD camera connected to a TV monitor. Cells in Layer V were targeted for filling by locating the YFP+ cells, which were faintly visible using the above filter combination (**Figure 1**). Individual neurons were targeted using transillumination and the contrast enhancement of the monitor. Cells were impaled and iontophoretically filled with dye using 3 ms square wave, 3 μA current pulses repeated at 0.5 Hz for 5–10 min delivered from a digital stimulator (Neuro Data PG4000) through an isolated current source (NeuroData SIU90). Neurons were filled in a sparse distribution along the slice (5–10 cells/hemisphere) to ensure that dendrites did not overlap (**Figure 1**). Instances in which tissue adhered to the pipette upon tip withdrawal were judged to be a result of poor fixation, typically resulting in dye diffusion out of the cell and/or removal of the nucleus. In these cases the cell was not used for further analysis. Once several cells were filled, the slice was post-fixed in 4% PFA pH 11 for 10 min at room temperature and stored in PBS with 0.01% sodium azide at 4°C prior to measurement.

### Immunohistochemistry and Counterstaining

All immunohistochemistry incubation steps were performed on floating slices at room temperature, on a shaker in the dark, with three PBS washes between each step and 0.01% sodium azide added for overnight incubations. All slices were permeabilized with 1% Triton X-100 in PBS for 2 h.

**FIGURE 2:**
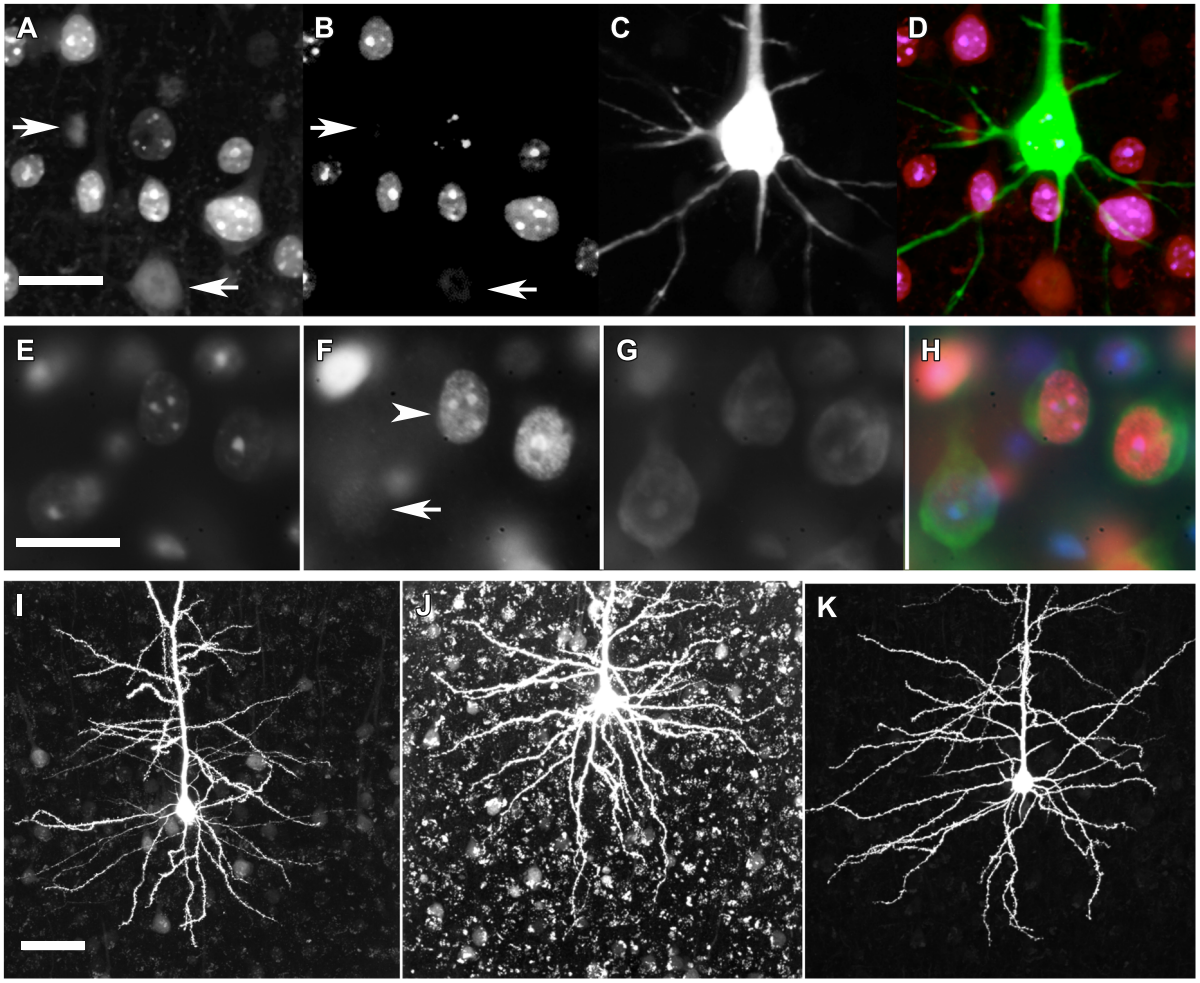
**Immunohistochemical genotyping to identify dye-filled, nuclear-, or cytoplasm-counterstained neurons for morphological comparisons. (A)** C-terminal MeCP2 antibody staining appears punctate in MeCP2+ neurons and diffuse in MeCP2- neurons (arrows). **(B)** N-terminal MeCP2 staining appears punctate in MeCP2+ neurons, and is absent in MeCP2- neurons (arrows). **(C)** Alexa Fluor 594 (AF594) iontophoretically injected into lightly fixed neurons fills the cell body and dendrites. **(D)** Composite image of **A–C**. Pink = MeCP2+, red = MeCP2-; in composite image the AF594 fluorescence obscures the punctate nuclear MeCP2+ staining that can be seen in panels **A** and **B** which show filled neuron is wild-type). Scale bar = 20 μm. **(E)** DAPI nuclear labeling reveals which cells are in the in focal plane and delineates the boundary of nucleus. **(F)** Punctate (arrowhead) or diffuse (arrow) staining of the C-terminal MeCP2 antibody differentiates MeCP2+ and MeCP2- cells, respectively. **(G)** The cytoplasm of neurons is labeled with Neurotrace Nissl body stain. **(H)** A composite image reveals the genotype of each neuron. MeCP2- cells appear blue/green because the red C-terminal MeCP2 stain is faint and diffuse. Scale bar = 20 μm. **(I)** Granular lipofuscin autofluorescence is faint in a brain slice at 9 months of age. **(J)** Lipofuscin accumulates in brain tissue over time. In an 18-months old animal strong autofluorescence makes the visualization and quantification of fluorescent dyes difficult. **(K)** Treatment of slices with 5 mM CuSO_4_ in 50 mM ammonium acetate considerably reduces lipofuscin autofluorescence without affecting AF594 staining. Scale bar = 50 μm.

Brain slices containing dye filled neurons were immunohistochemically labeled to detect the genotype of individual neurons (**Figure 2**). Slices were blocked with 5% normal goat serum in PBS overnight, then incubated overnight with monoclonal mouse anti-N terminal MeCP2 (Sigma 4B6) and monoclonal chicken anti-C terminal MeCP2 (Millipore ABE171) primary antibodies prepared at 0.2 μg/ml in 1% normal goat serum and PBS. Primary antibodies were detected with goat anti-mouse conjugated to Alexa Fluor 647 (Invitrogen) and goat anti-chicken conjugated to Alexa Fluor 488 (Invitrogen) secondary antibodies (4 μg/ml) prepared in 1% normal goat serum in PBS and incubated overnight. Slices were counterstained for 10 min with 30 nM 4^′^,6-diamidino-2-phenylindole (DAPI) in PBS.

Brain slices prepared for soma and nuclear size analysis (**Figure 2**) were incubated overnight with monoclonal chicken anti-C terminal MeCP2 (Millipore ABE171) primary antibody prepared at 0.2 μg/ml in PBS. The primary antibody was detected using goat anti-chicken secondary antibodies conjugated to Alexa Fluor 555 (Invitrogen) prepared at 4 μg/ml in PBS and incubated overnight. Slices were counterstained with DAPI (30 nM) and the deep-red fluorescent Nissl stain Neurotrace 640/660 (Invitrogen) overnight.

### Lipofuscin Reduction

Broad-spectrum lipofuscin autofluorescence in older mice interferes with the visibility and quantification of neuronal dendrites (**Figure 2**). After completing immunohistochemistry and counterstaining protocols, lipofuscin autofluorescence was quenched by treating cortical slices with 5 mM CuSO_4_ in 50 mM ammonium acetate for 10 min (Schnell et al., 1999).

### Fluorescence Microscopy

Slices were mounted on poly-l-lysine coated slides and covered with #1.5 coverslips using Immu-mount medium (Thermo Scientific). All images for quantification of dendrite structure were obtained using an Olympus UPlanFLN 40X/1.3 NA oil objective. Confocal stacks of single cells microinjected with Alexa Fluor 594 hydrazide were acquired using a 543 nm laser, 610/100 emission filter, 640 × 640 pixel resolution, 0.5 μm steps, 40 μs/pixel dwell, and high laser power. The high voltage, gain, and offset were modified during imaging to optimize visibility of the dendrites and ensure that the end of each dendrite was clearly ascertained. Any dendrites that projected beyond the field of view were captured in an additional adjacent stack. In order to genotype individual neurons, a separate confocal stack centered on the cell body was acquired at 6X zoom with additional channels for detection of the C-terminal (488 nm laser, 515/20 emission) and N-terminal (635 nm laser, 705/100 emission) MeCP2 antibodies. Brain slices prepared for soma and nuclear size analysis were viewed on an Olympus IX70 inverted epifluorescence microscope. 5–20 images per animal were captured with a Retiga 2000R digital CCD camera (QImaging, Surrey, BC, Canada) with an additional 1.6X post objective magnification.

### XCI Ratios and Soma/Nuclear Size Measurements

Widefield fluorescence images were obtained from slices of tissue from 30 *Mecp2*^+/-^ animals in one experiment and 11 *Mecp2*^+/-^ and 10 *Mecp2*^+/+^ animals in a second experiment (30–80 cells/animal). Images were obtained using a UPlanFLN 40X 1.30 NA oil immersion objective (Olympus) focused at 2–3 separate planes, between 10 and 25 μm below the surface, each separated by ∼5 μm. Inclusion criteria for measured cells at each focus position were: (1) the DAPI-stained nucleus must be in sharp focus in the focal plane (2) the Neurotrace-stained soma must not be occluded by overlying cells, and (3) the genotype must be clearly identifiable using C-terminal MeCP2 antibody staining. MeCP2+ neurons were counted if punctate staining of the C-terminal antibody (colocalized with the DAPI staining) was visible. MeCP2- neurons were counted if DAPI and diffuse C-terminal antibody staining were visible. Outlines of somata (Neurotrace) and nuclei (DAPI) were manually traced at 8X image magnification using ImageJ (Abràmoff et al., 2004). All in-focus neurons that were completely within the field of view and met the above criteria were measured and since X-inactivation is skewed toward wild-type more MeCP2+ than MeCP2- neurons were measured.

### Neuronal Reconstruction and Morphological Analysis

**FIGURE 3:**
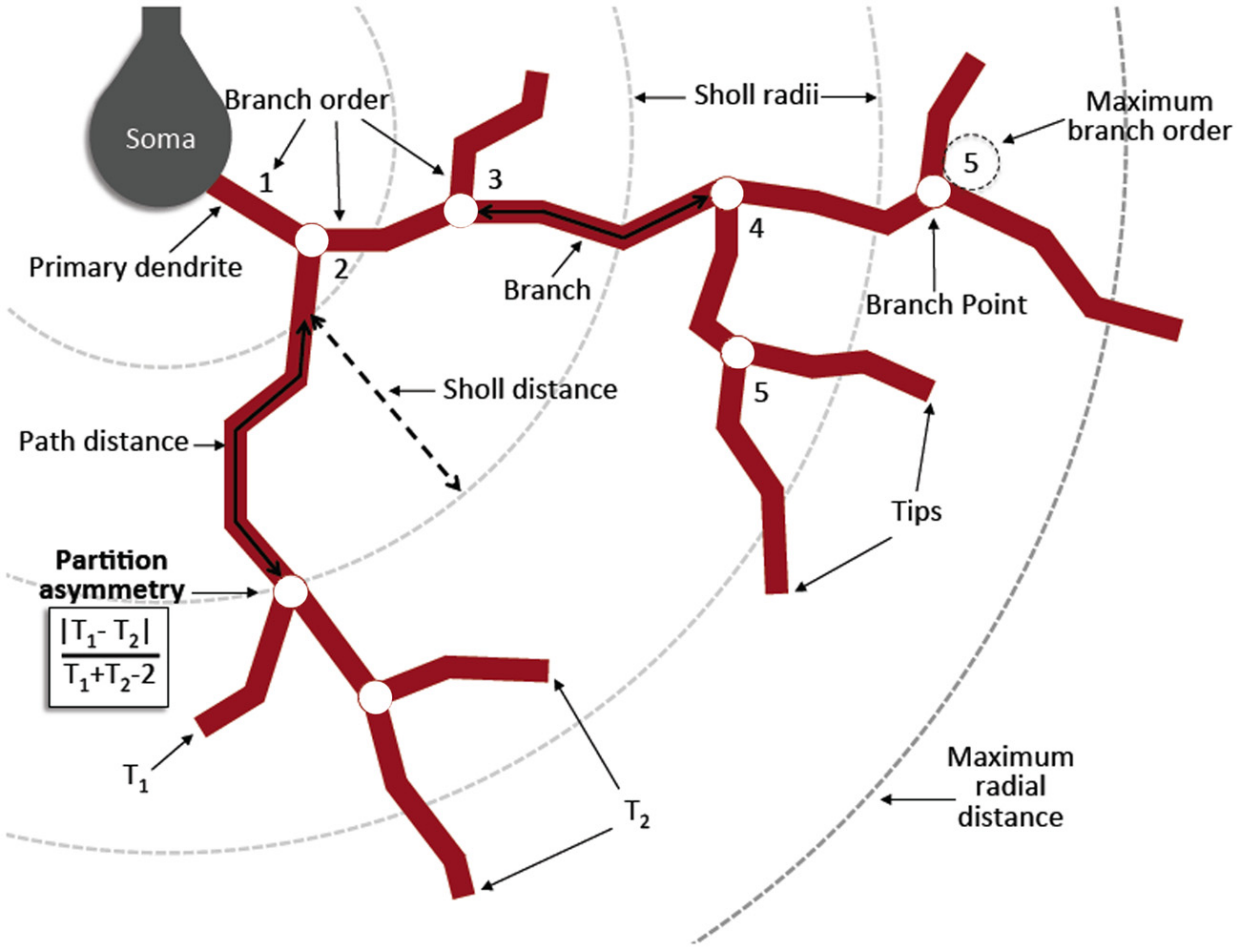
**Diagram of morphometric measurements.** A *primary dendrite* is any dendrite originating from the cell body. A *branch point* occurs when the original dendrite bifurcates into two or more daughter trees. A centrifugal labeling scheme was used for assigning *branch order*, where the lowest order is given to the primary dendrites and increases when a branch point is reached. *Maximum branch order* is the maximum value reached by any primary tree on a given cell. A *branch* is a length of dendrite between the soma and a branch point, between two branch points, or between a branch point and a tip. A *tip* is the termination of any branch. *Sholl radii* are concentric spheres originating from the centroid of the soma and increasing at 10 μm intervals. *Maximum radial distance* is the maximum Sholl radius reached by any single dendrite per cell. *Partition asymmetry* is calculated at each branch point as | T_1_–T_2_| /(T_1_ + T_2_-2) where T_1_ and T_2_ are the number of tips of the two daughter trees, with values of one and zero indicating asymmetrical and symmetrical partitioning respectively. *Contraction* is calculated for each branch as a ratio between the Euclidean distance and the distance along the branch path. A value of one indicates perfectly straight dendrites, while lower values indicate increasing levels of tortuosity or winding of the dendrites. Derived in part from (Costa et al., 2010).

Eighty two MeCP2 WT neurons, 98 MeCP2+ and 59 MeCP2- neurons were filled and reconstructed (five animals per genotype, 16–31 cells per animal). In order to minimize variation associated with poor fills or chopped dendrites neurons were excluded if: (1) the genotype could not be determined (2) less than three primary dendrites were intact (3) more than half of the total primary dendrites on the cell body were chopped off by the slicing (4) tips of the dendrites were not visible (5) an apical dendrite of at least 75 micrometers length was not visible. If dendrites extended beyond the edge of one field of view additional image stacks were obtained and tiled in 3D using the Volume Integration and Alignment System (VIAS; Rodriguez et al., 2003). ImageJ was used to adjust the image brightness/contrast and images were saved as 8-bit tiffs. 3D neuronal reconstructions were generated in Neuronstudio (Wearne et al., 2005) using a Cintiq 21q tablet. Neuron tracings were exported in SWC file format, for compatibility with standard morphometric software tools. Multiple morphological parameters were extracted using the freeware applications Neuronstudio, Simple Neurite Tracer (Longair et al., 2011), and L-Measure (Scorcioni et al., 2008). The morphological parameters chosen for analysis in this study are listed in **Table 2** and further described in **Figure 3** adapted from procedures and conventions described by Scorcioni et al. (2008) and Costa et al. (2010).

**Table 2:** Morphological parameters analyzed from 3D neuronal reconstructions.

Program	Parameter/description
Neuronstudio	Sholl length (μm) – Total length of all branches per 10 μm Sholl radius
	Cumulative Sholl length (μm) – Cumulative length of all branches per 10 μm Sholl radius
	Sholl branch points – Total number of branch points per 10 μm Sholl radius
	Branch length/branch order – Average branch length per branch order
L-Measure	Total number of primary dendrites stemming from the soma
	Total length of all branches per branch order
	Number of branch points per branch order
	Number of branches per branch order
	Number of tips per branch order
	Maximum radial distance (μm) – Euclidean distance between the soma and the farthest compartment reached by a dendrite
	Maximum branch order per 10 μm Sholl radius
	Contraction per branch order – ratio between the Euclidean distance and branch path distance calculated per branch order
	Partition asymmetry – where T_1_ and T_2_ are the number of tips of each daughter tree. Computed at every branch point and plotted as function of branch order

### Statistical Analysis

Statistical analyses were conducted using Prism software (GraphPad^TM^). Data are presented as mean ± standard error of the mean (SEM). All parameters assessed across Sholl radii (Sholl, 1953) or branch orders were analyzed using two-way repeated measures ANOVA with multiple comparisons made using the Bonferroni post-test. Primary dendrite number was assessed using a one-way ANOVA and multiple comparisons were made using the Bonferroni post-test. Direct comparisons of soma and nuclear areas between animals of different genotypes were performed using the non-parametric two-tailed Mann–Whitney *U* test. Comparisons between cells of different genotypes within the same animal were performed using the paired Wilcoxon signed rank test. Linear regressions were used to compare the slopes and y-intercepts of soma and nuclear area data obtained across ages and XCI ratios. Non-linear regressions were fit to the frequency distributions of soma and nuclear areas and the best-fit values were compared. *p*-values of ≤0.05 were considered significant for all statistical tests.

## Results

### Sholl Analysis of Basal Dendritic Arbor of Motor Cortical Layer V Pyramidal Neurons of MeCP2 WT, MeCP2+, and MeCP2- Neuronal Genotypes

**FIGURE 4:**
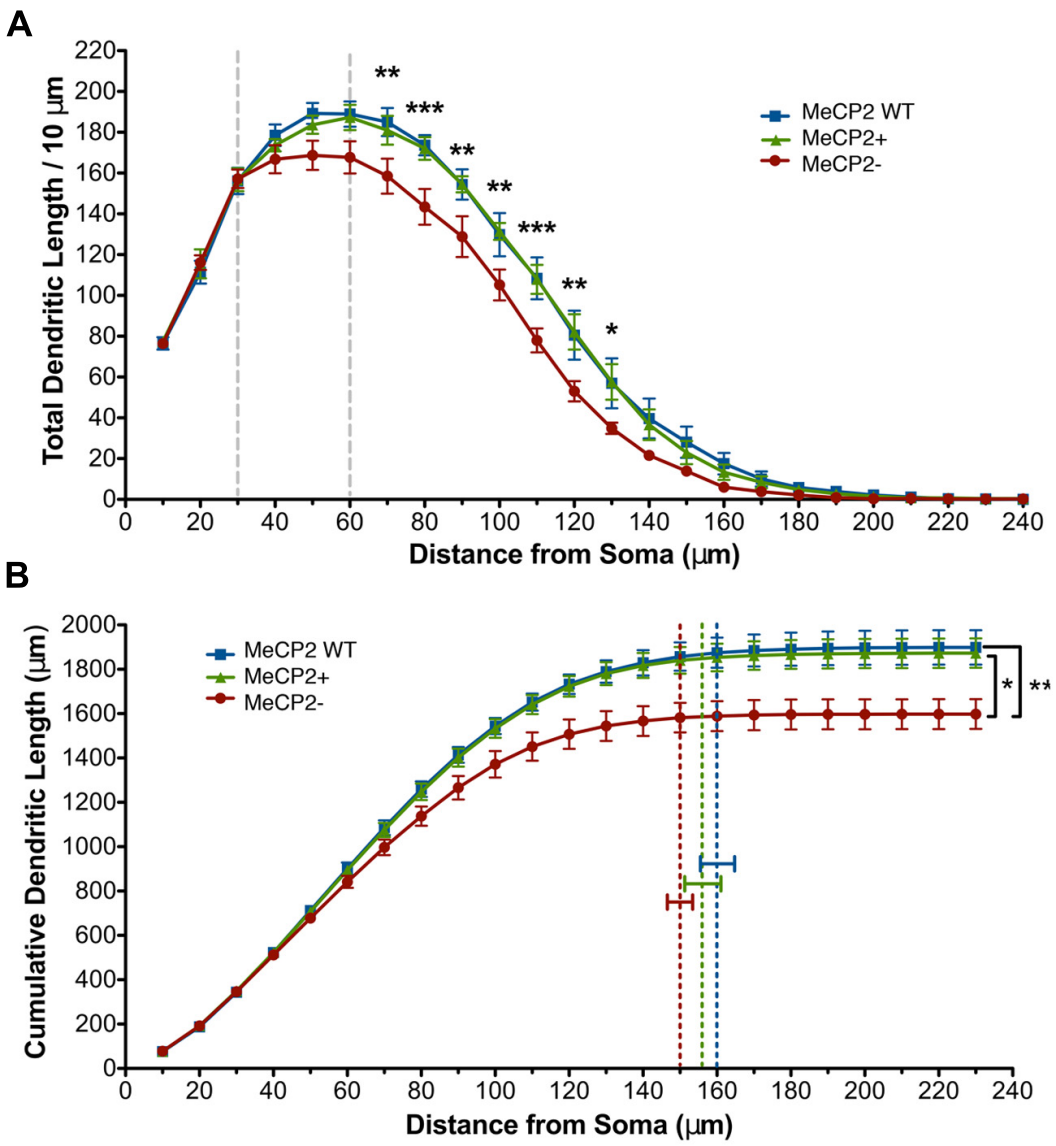
**Total dendritic length of MeCP2- neurons is reduced. (A)** MeCP- neurons have decreased dendritic length at distances of 70–130 μm from the soma compared to both MeCP2 WT and MeCP2+ neurons. The region between the dashed lines indicates where the slopes of MeCP2- and MeCP2+ cells deviate. **(B)** MeCP2- neuron total basal dendritic length is 15% less (275 μm) compared to both MeCP2 WT and MeCP2+ neurons. Dashed lines indicate the average maximum radial distance reached for each genotype. No significant difference was found between the genotypes in this measure. ^∗^*p* < 0.05, ^∗∗^*p* < 0.01, and ^∗∗∗^*p* < 0.001. For **Figures 4–6**, 82 MeCP2 WT neurons, 98 MeCP2+ and 59 MeCP2- neurons were filled and reconstructed (five animals per genotype, 16–31 cells per animal).

Sholl analysis (Sholl, 1953) was used to determine how a mutation in *Mecp2* affects the dendritic morphology of MeCP2+ and MeCP2- neurons compared to MeCP2 WT neurons. The total dendritic length per 10 μm Sholl radius was reduced in MeCP2- neurons at distances of 70–130 μm from the soma compared to both MeCP2 WT and MeCP2+ neurons (two-way repeated measures ANOVA and Bonferroni post-tests, *F*_(62,372)_ = 2.33, *p* < 0.0001; **Figure 4A**). MeCP2 WT and MeCP2+ neurons are not different at any Sholl radius (*p* > 0.05). The slopes of the MeCP2+ and MeCP2 WT curves diverge from that of the MeCP2- curve between 30 and 60 μm from the cell body, and run parallel at greater distances, indicating that differences in length between the genotypes are initiated in this region and maintained into the distal regions.

To quantify the total differences in dendritic length observed in **Figure 4A**, cumulative dendritic length was calculated at each successive 10 μm Sholl radius (**Figure 4B**). MeCP2- neurons have a significant reduction (15% or 275 μm) in cumulative dendritic length compared to both other genotypes, while MeCP2 WT and MeCP2+ are not significantly different [MeCP2- = 1600.6 ± 66.2 μm, MeCP2 WT = 1898.5 ± 77.6 μm, MeCP2+ = 1873.9 ± 66.5 μm; two-way repeated measures ANOVA and Bonferroni post-tests, *F*_(44,264)_ = 4.68, *p* < 0.0001]. The maximum radial distance was not different between the genotypes [MeCP2- = 149.7 ± 4.3 μm, MeCP2 WT = 159.7 ± 5.9 μm, MeCP2+ = 155.5 ± 6.2 μm; one-way ANOVA, *F*_(2,12)_ = 0.83, *p* = 0.5] suggesting that although MeCP2- neurons have less length than MeCP2+ neurons, the reach of their dendritic arbor is not significantly lessened.

**FIGURE 5:**
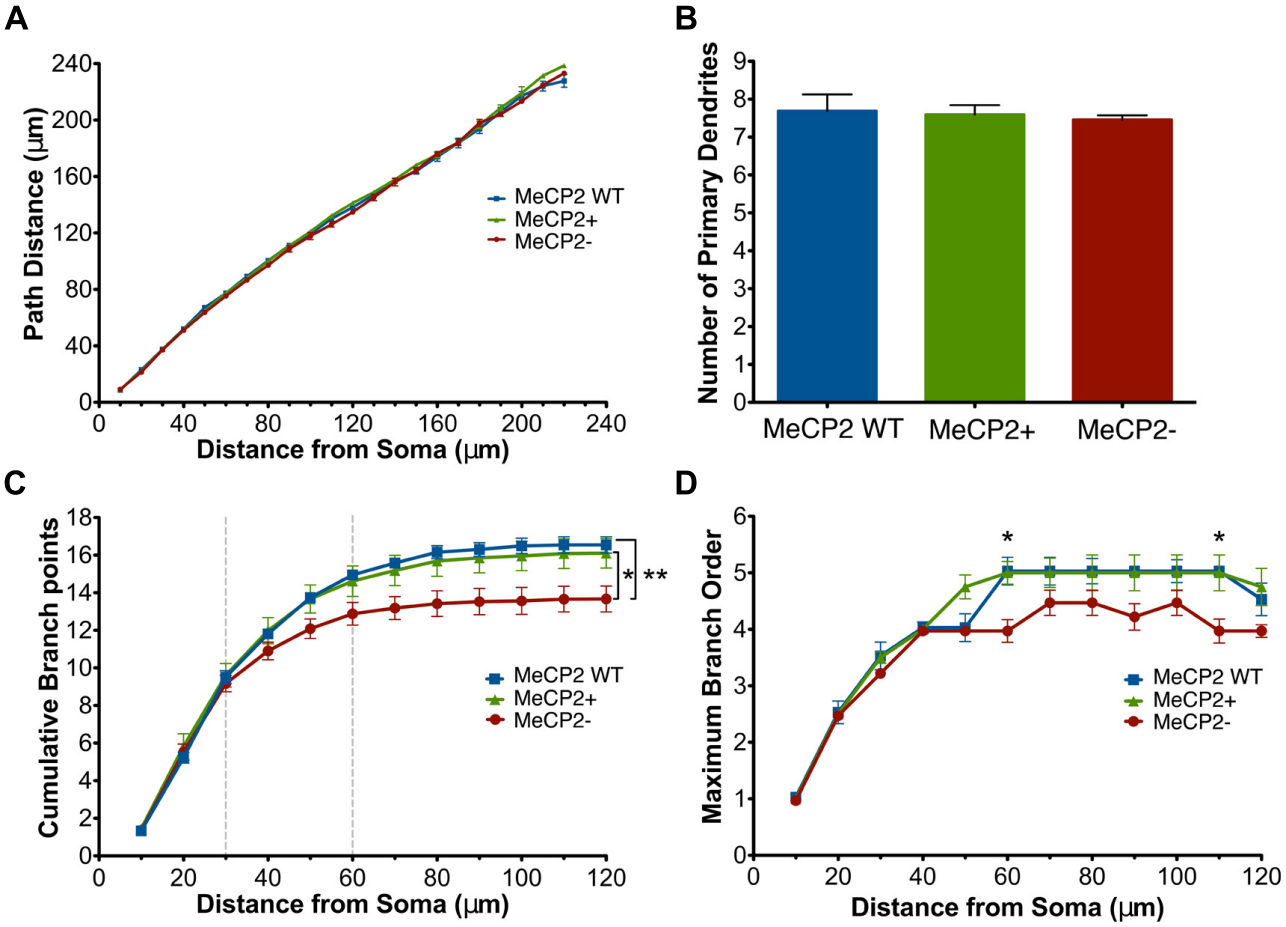
**MeCP2- neurons have a reduction in the number of total branch points and maximum branch order across Sholl radii. (A)** Basal dendrites of all genotypes have similar path distances across Sholl radii. **(B)** All genotypes have the same number of primary dendrites. **(C)** MeCP2- neurons have an average of three fewer total branch points (a 17% reduction) compared to both other genotypes. The region between the dashed lines indicates where the MeCP2- neurons differ from MeCP2+ neurons in trend. **(D)** The maximum branch order reached by MeCP2- neurons at distances of 60 and 110 μm from the soma is lower than both other genotypes. ^∗^*p* < 0.05 and ^∗∗^*p* < 0.01.

Branch path distances at successive 10 μm Sholl radii were compared across genotypes (**Figure 5A**) to determine whether the cumulative length deficiency of MeCP2- neurons is due to a decreased tortuosity of the branches. No differences were found between the genotypes at any Sholl radii [two-way repeated measures ANOVA and Bonferroni post-tests, *F*_(30,180)_ = 1.05, *p* = 0.4], indicating that the reduction in MeCP2- dendritic length is not due to straighter branches.

The number of primary dendrites was compared (**Figure 5B**) across neuronal genotypes to determine if the differences in length of MeCP2- neurons (**Figure 4B**) could be accounted for by their having a different number of primary branches. There was no difference in the total number of primary dendrites across all genotypes [one-way ANOVA, *F*_(2,12)_ = 0.15, *p* = 0.9].

Examining the cumulative number of branch points at each successive 10 μm Sholl radius (**Figure 5C**) revealed that MeCP2- neurons have an average of three (17%) fewer total branch points than both other genotypes [MeCP2- = 13.7 ± 0.7, MeCP2 WT = 16.5 ± 0.4, MeCP2+ = 16.2 ± 0.8; *t*_(24)_ = 3.51, *p* < 0.01, two-way repeated measures ANOVA and Bonferroni post-tests, *F*_(24,144)_ = 8.58, *p* < 0.0001]. The slopes of the lines differ between 30 and 60 μm from the soma, suggesting that MeCP2- neurons have fewer branch points in this region.

The reduced number of branch points in MecP2- cells could be the result of either fewer simple primary trees (low maximum branch order), fewer complex ones (high maximum branch order), or both. In order to determine which was the case, the maximum branch order reached per cell at each successive 10 μm Sholl radii was compared across the genotypes (**Figure 5D**). At 60 μm (MeCP2- = 4 ± 0.20, MeCP2 WT = 5 ± 0.3, MeCP2+ = 5 ± 0.2; *t*_(24)_ = 3.43, *p* < 0.05) and 110 μm (MeCP2- = 4 ± 0.2, MeCP2 WT = 5 ± 0.1, MeCP2+ = 5 ± 0.3; *t*_(24)_ = 3.43, *p* < 0.05) from the soma MeCP2- neurons have a lower maximum branch order compared to both other genotypes [two-way repeated measures ANOVA and Bonferroni post-tests, *F*_(2,12)_ = 1.38, *p* < 0.0001]. Since MeCP2+ cells have a relatively high maximum branch order (five) at these distances, this suggests that MeCP2- cells have fewer overall branch points because they have fewer complex branches.

### Branch Order Analysis of MeCP2 WT, MeCP2+, and MeCP2- Neuronal Genotypes

**FIGURE 6:**
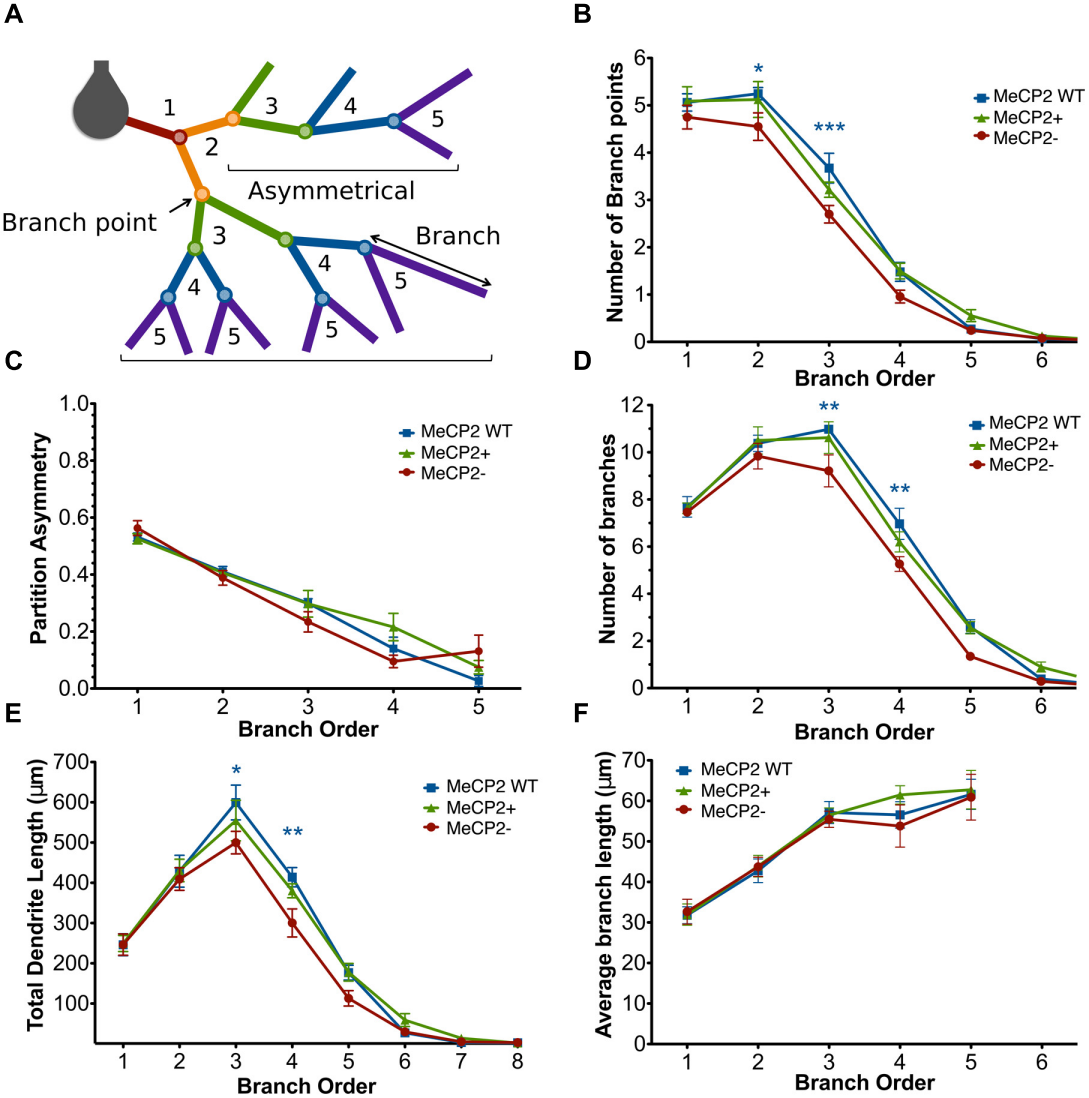
**MeCP2- neurons have fewer third and fourth order branches compared to both other genotypes. (A)** Branch order diagram depicting the increasing order values with each successive branch point from the cell body. Symmetrical and asymmetrical branching patterns are shown (calculated at the second order branch points) which result in partition asymmetry values of 0 and 1, respectively. **(B)** MeCP2- neurons have fewer second and third order branch points compared to both genotypes. **(C)** Branching patterns in MeCP2- neurons are unaltered. **(D)** MeCP2- neurons have fewer third and fourth order branches. **(E)** MeCP2- neurons have reduced dendritic length in the third and fourth branch orders. **(F)** The average length of MeCP2- branches per branch order does not differ from that of MeCP2+ branches. ^∗^*p* < 0.05, ^∗∗^*p* < 0.01 and ^∗∗∗^*p* < 0.001.

Branch order analysis was used to determine how a reduction in total branch point number affects the detailed branch structure of Mecp2- neurons (**Figure 6**). The number of branch points within each branch order was first compared across the neuronal genotypes (**Figure 6B**). MeCP2- neurons have fewer branch points at the second [MeCP2- = 4.6 ± 0.3, MeCP2 WT = 5.3 ± 0.1, MeCP2+ = 5.1 ± 0.4; *t*_(16)_ = 3.05, *p* < 0.05] and third [MeCP2- = 2.7 ± 0.2, MeCP2 WT = 3.7 ± 0.3, MeCP2+ = 3.2 ± 0.2; *t*_(16)_ = 4.25, *p* < 0.001] branch orders compared to both other genotypes [two-way repeated measures ANOVA and Bonferroni post-tests, *F*_(16,96)_ = 1.69, *p* = 0.03]. MeCP2- neurons therefore have, on average, one less branch point at each of the second and third branch orders. No statistically significant differences were found between MeCP2- and MeCP2+ cells at the first or fourth branch orders, yet MeCP2- cells appear to have fewer branch points here, raising the possibility that these branch orders may be affected by *MeCP2* mutation as well.

Partition asymmetry was next calculated at each branch order to assess whether branching patterns were altered in MeCP2- neurons (**Figure 6C**). No difference was found between the genotypes across branch orders [two-way repeated measures ANOVA and Bonferroni post-tests, *F*_(8,48)_ = 2.23, *p* = 0.58), indicating that MeCP2- neurons do not have more asymmetric branching patterns than MeCP2+ cells.

The number of branches per branch order was then calculated (**Figure 6D**) to confirm that the reduced numbers of second and third order branch points seen in MeCP2- neurons also results in a reduction in the number of subsequent branches in the third and fourth branch orders of these neurons. MeCP2- neurons have 16% fewer third order branches [∼2 branches; MeCP2- = 9.2 ± 0.7, MeCP2 WT = 11.0 ± 0.1, MeCP2+ = 10.6 ± 0.7; *t*_(17)_ = 3.59, *p* < 0.01] and 25% fewer fourth order branches [∼2 branches; MeCP2- = 5.3 ± 0.3, MeCP2 WT = 7.0 ± 0.7, MeCP2+ = 6.2 ± 0.4; *t*_(14)_ = 3.46, *p* < 0.01; two-way repeated measures ANOVA and Bonferroni post-tests, *F*_(14,84)_ = 1.51, *p* = 0.02]. These data confirm that the lesser numbers of second and third order branch points result in a reduction in third and fourth order branches.

The total dendritic length per branch order was quantified (**Figure 6E**) to see if the reduced number of third and fourth order branches in MeCP2- neurons could account for the total reduction in dendritic length observed in these cells. MeCP2- neurons were found to have significantly less length in the third (17%) and fourth (27%) branch orders, as was predicted from the reduced number of third and fourth order branches [third order: MeCP2- = 499.8 ± 28.0 μm, MeCP2 WT = 599.5 ± 43.2 μm, MeCP2+ = 554.6 ± 50.4 μm; *t*_(14)_ = 2.93, *p* < 0.05; fourth order: MeCP2- = 300.3 ± 34.9 μm, MeCP2 WT = 414.0 ± 23.9 μm, MeCP2+ = 380.2 ± 17.4 μm; *t*_(14)_ = 3.34, *p* < 0.01; two-way repeated measures ANOVA and Bonferroni post-tests, *F*_(14,84)_ = 1.15, *p* = 0.03]. These data indicate that MeCP2- cells have 100 and 115 μm less dendritic length in the third and fourth branch orders, respectively.

Finally, the average dendritic length per branch order was assessed (**Figure 6F**) to determine whether the shorter branches observed in MeCP2- neurons could also contribute to the reduced length observed in these cells. No difference was found in average branch length per branch order between the genotypes [two-way repeated measures ANOVA and Bonferroni post-tests, *F*_(8,48)_ = 0.36, *p* = 0.8]. This suggests that the shorter dendritic length observed in MeCP2- neurons is not caused by their having shorter branches than the other genotypes, but primarily from their reduced number of third and fourth order branches.

### Cell Autonomous Effects on Soma and Nuclear Areas

**FIGURE 7:**
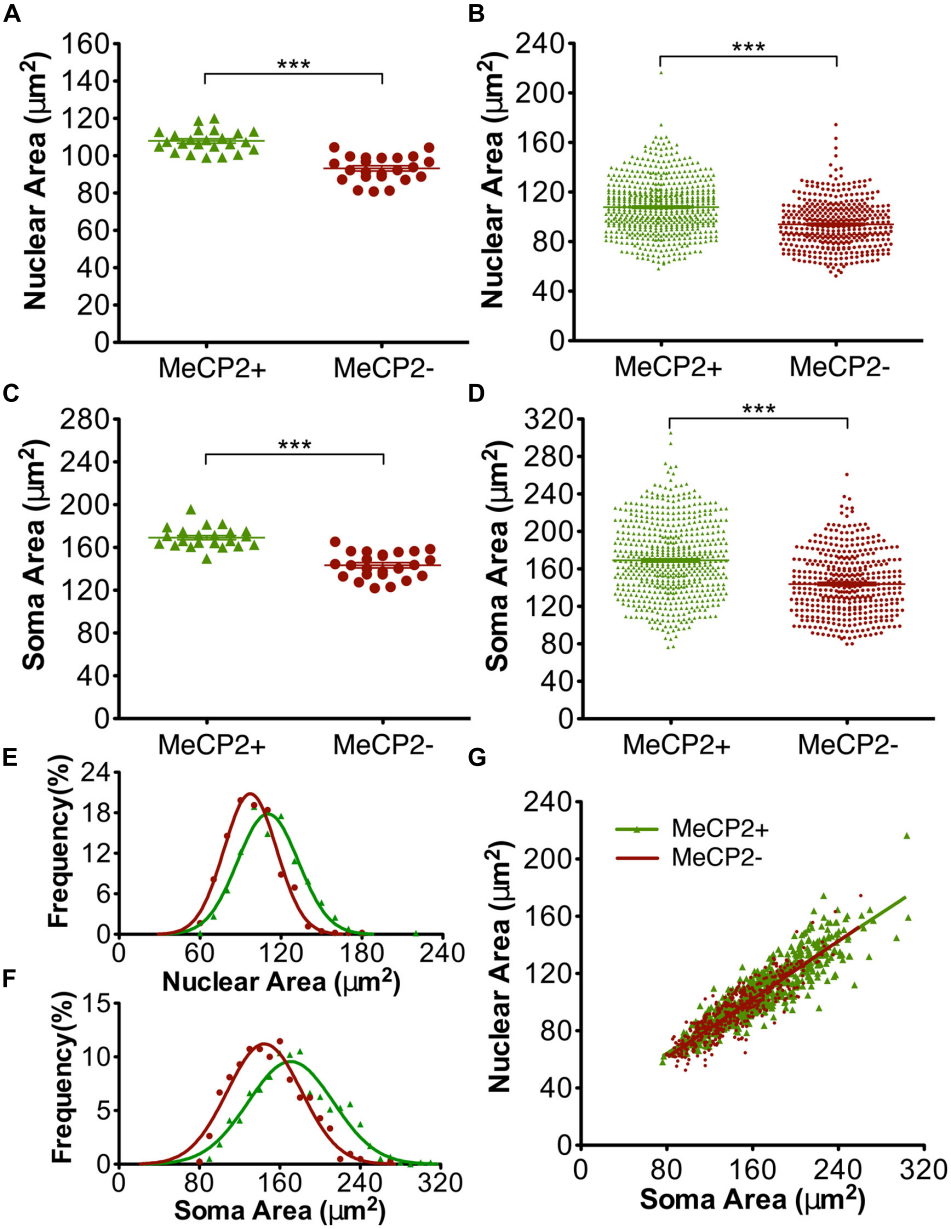
**MeCP2- neurons have smaller somata and nuclei than MeCP2+ neurons.** MeCP2- nuclei **(A)** and somata **(C)** are on average 13 and 15% smaller than MeCP2+ nuclei and somata, respectively (*n* = 24 animals). The population of MeCP2- nuclei **(B)** and somata **(D)** have fewer large nuclei and somata compared to MeCP2+ (MeCP2+ *n* = 598 cells and MeCP2- *n* = 418 cells). Frequency distributions of nuclear **(E)** and soma **(F)** areas are significantly different between the genotypes (*p* < 0.0001, nuclear area MeCP2+ *R*^2^ = 0.96 and MeCP2-*R*^2^ = 0.98; Soma area MeCP2+ *R*^2^ = 0.93 and MeCP2-*R*^2^ = 0.94). **(G)** Soma area is a predictor of nuclear area in both genotypes. Slopes are equal between the genotypes (*p* = 0.30, MeCP2+ *R*^2^ = 0.79 and MeCP2- *R*^2^ = 0.78). ^∗∗∗^*p* < 0.001.

The nuclear and somatic areas of MeCP2+ and MeCP2- Layer V motor pyramidal neurons were measured in *Mecp2^+/-^* mice (**Figure 7**). The mean nuclear area of MeCP2- neurons in these experiments was 13% smaller than that of MeCP2+ neurons [*n* = 24 animals, MeCP2- = 93.3 ± 1.4 μm^2^, MeCP2+ 107.9 ± 1.2 μm^2^, *t*_(46)_ = 8.04, *p* < 0.0001; **Figure 7A**]. A plot of the entire population of individual nuclear areas [MeCP2+ *n* = 598 neurons, MeCP2-*n* = 418 neurons (**Figure 7B**)] shows that the proportion of small neurons between the two genotypes are comparable, while the proportion of neurons in the largest size class is considerably smaller in the MeCP2- cell population than it is in the MeCP2+ population. The mean soma area of MeCP2- neurons is 15% smaller than MeCP2+ [*n* = 24 animals, MeCP2- = 143.3 ± 2.5 μm^2^, MeCP2+ 169.2 ± 1.9 μm^2^, *t*_(46)_ = 8.25, *p* < 0.0001; **Figure 7C**]. A graph of individual soma areas (MeCP2+ *n* = 598 neurons, MeCP2-*n* = 418 neurons, **Figure 7D**) shows that smallest somata of MeCP2- (79.8 μm^2^) and MeCP2+ (76.3 μm^2^) genotypes are comparable, while the largest quartile of MeCP2- somata is considerably smaller than the largest quartile of MeCP2+ somata. Frequency distributions of the areas of nuclei (**Figure 7E**) and somata (**Figure 7F**) show that the distribution of MeCP2- neuron sizes is significantly shifted toward the smaller size classes compared to MeCP2+ neurons [nuclear area *t*_(3,21)_ = 23.66, *p* < 0.0001, soma area *t*_(3,36)_ = 27.57, *p* < 0.0001]. To assess whether the soma or nuclear areas in MeCP2- neurons were reduced disproportionally in relation to the each other, MeCP2+ and MeCP2- soma areas were plotted against their respective nuclear areas from within the same population (**Figure 7G**). The resulting slopes of MeCP2- and MeCP2+ populations were not significantly different [*F*_(1,1012)_ = 1.08, *p* = 0.3] showing that while MeCP2- nuclei and soma are smaller in area than their MeCP2+ counterparts, the proportion of soma and nuclear areas are similar in both genotypes.

### Symptom Severity of Mecp2^+/-^ Mice Across Age and XCI Ratios

**FIGURE 8:**
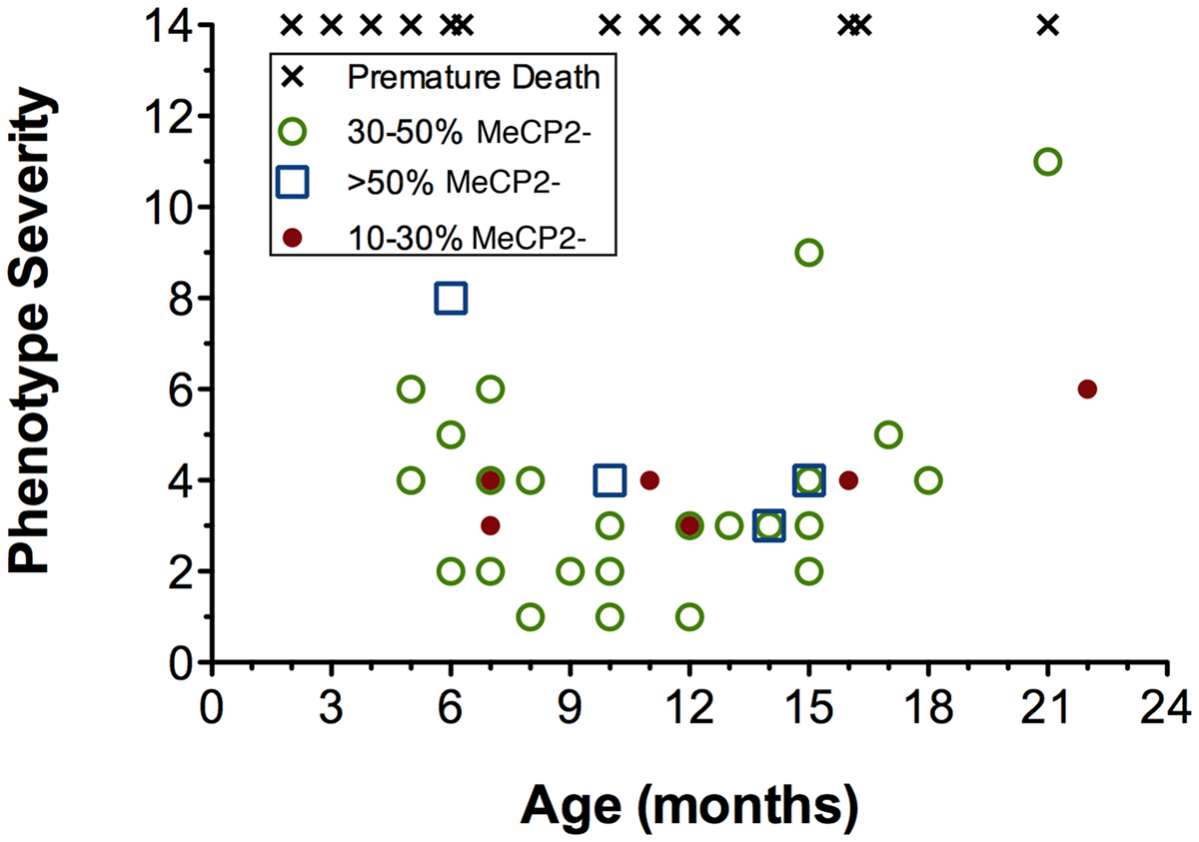
**Age, phenotype severity and XCI ratios are not correlated in *Mecp2^+/-^* mice.** Increasing age is not a predictor of phenotype severity of *Mecp2^+/-^* mice. Aged *Mecp2^+/-^* animals are not more likely to have highly skewed XCI ratios (10–30% mutant neurons, red circles) and XCI ratios do not predict phenotype severity at any age. Premature death (x’s) occurred across both young and old age groups. A premature death is defined as any animal that died spontaneously (not sacrificed for experiments), therefore XCI ratios were unavailable.

To determine the interaction between age, cell phenotype, and XCI ratios, animals were scored for their phenotype severity at their time of sacrifice for immunohistochemical staining (from 5 to 22 months of age). Within the cohort of mice selected for our study the phenotype severity was highly variable, with some mice dying prematurely as young as 2 months of age (i.e., before they could be used for experiments), and others surviving into old age (22 months) with only mild symptoms (**Figure 8**). Increasing age did not correlate with increased phenotype severity (*p* = 0.2, *R*^2^ = 0.038). To determine whether symptom levels vary according to XCI ratio, XCI ratios were analyzed by immunohistochemistry and compared against phenotype severity. It was found that XCI ratios were also not correlated with phenotype severity (**Figure 8**; *p* = 0.66, *R*^2^ = 0.0062). Surprisingly, mice with a highly skewed XCI ratio favoring expression of the wild-type chromosome (10% MeCP2-:90% MeCP2+) did not have less severe symptoms than mice with a more balanced XCI ratio (50% MeCP2-:50% MeCP2+).

### Effect of Age on Soma and Nuclear areas

**FIGURE 9:**
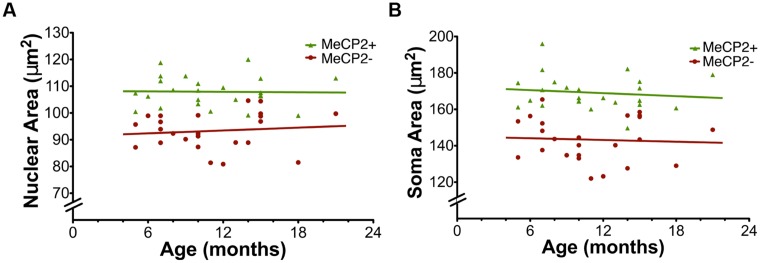
**Age does not affect nuclear and soma areas. (A)** Nuclear area in MeCP2+ and MeCP2- neurons is not altered across age (MeCP2+ *R*2 = 0.00040 and MeCP2-*R*^2^ = 0.012). **(B)** Soma area in both genotypes is not affected by age (MeCP2+ *R*2 = 0.015 and MeCP2-*R*2 = 0.0031).

Age did not affect nuclear area [MeCP2+ *F*_(1,22)_ = 0.0089, *p* = 0.92, MeCP2- F_(1,22)_ = 0.012, *p* = 0.61] or somatic area [MeCP2+ *F*_(1,22)_ = 0.34, *p* = 0.57, MeCP-*F*_(1,22)_ = 0.069, *p* = 0.79] for either genotype (**Figure 9**). The linear regression slopes for MeCP2+ and MeCP2- cells are not significantly different in either nuclear [*F*_(1,44)_ = 0.21, *p* = 0.65] or soma [*F*_(1,44)_ = 0.023, *p* = 0.88] areas, plotted across age from 5 to 22 months.

### Effect of XCI Ratios on Soma and Nuclear Areas

**FIGURE 10:**
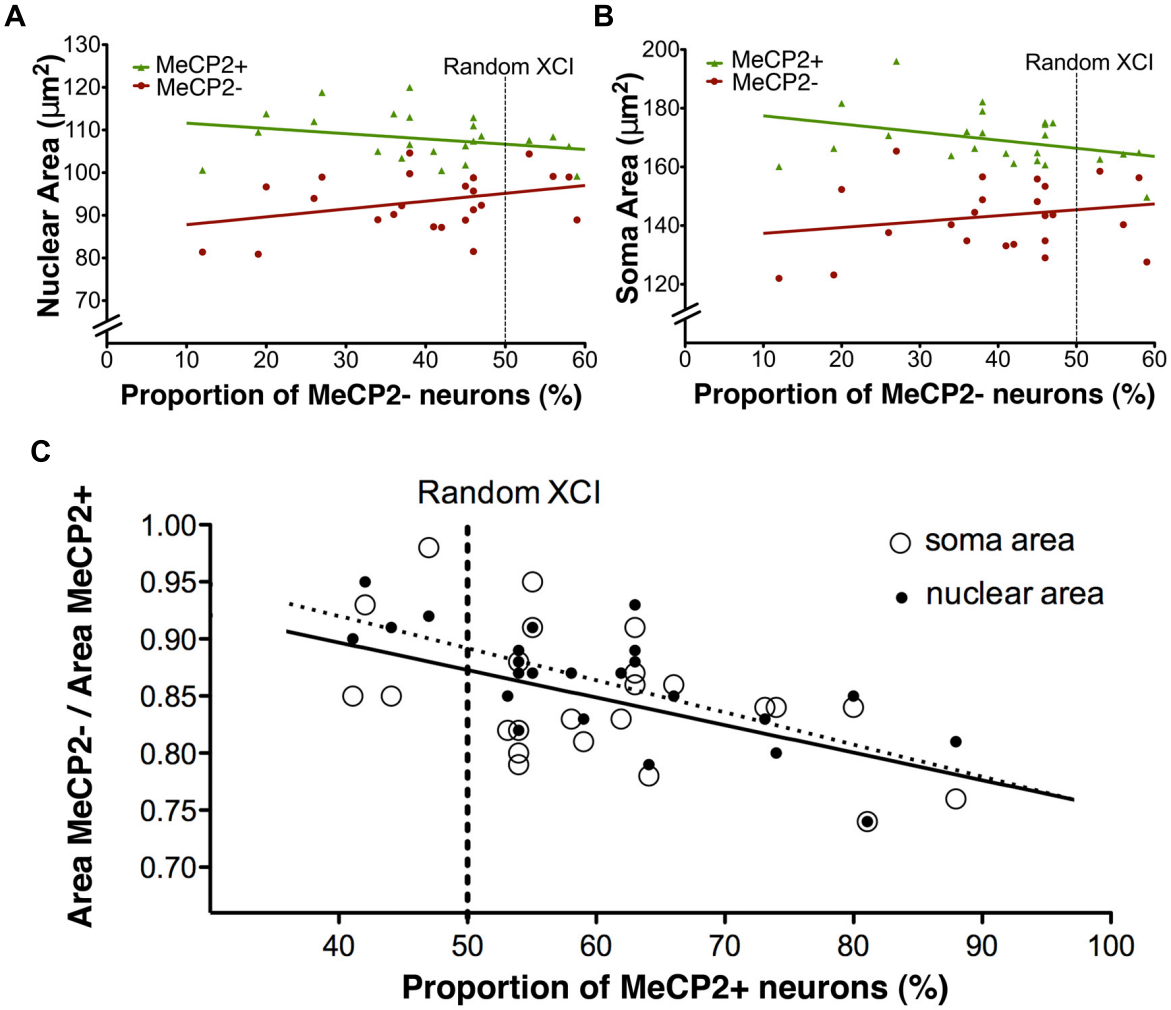
**Nuclear and soma areas are correlated with XCI ratios. (A)** Nuclear areas of MeCP2+ and MeCP2- neurons diverge as XCI ratios become more skewed in favor of MeCP2+ expression (right to left). Slopes are significantly different between the genotypes (*p* = 0.04, MeCP2+ *R*^2^ = 0.069 and MeCP2- *R*^2^ = 0.11). **(B)** Soma areas of MeCP2+ and MeCP2- neurons appear to diverge as the proportion of MeCP2- neurons in the brain decreases (right to left). Slopes are not significantly different between the two genotypes (*p* = 0.07, MeCP2+ *R*^2^ = 0.13 and MeCP2- *R*^2^ = 0.041). **(C)** MeCP2- soma and nuclear areas were normalized to paired MeCP2+ values from the same animal. MeCP2- nuclei and somata in brains with XCI ratios highly skewed toward wild-type (>70%% MeCP2+) are significantly smaller than MeCP2- nuclei and somata in brains with more balanced XCI ratios (∼50% MeCP2+). Slopes are significantly non-zero (nuclear area *p* = 0.0003 *R*^2^ = 0.45, soma area *p* = 0.0095, *R*^2^ = 0.29).

To determine if XCI ratios have an effect on soma or nuclear sizes of MeCP2+ and MeCP2- neurons, somatic and nuclear areas were plotted against the proportion of MeCP2- neurons in the brain (**Figure 10**). The linear regression slope of MeCP2- nuclear area was significantly different from that of MeCP2+ nuclear area [F_(1,44)_ = 4.26, *p* = 0.045; **Figure 10A**]. This indicates that as the proportion of wild-type neurons in the brain increases, the nuclear area of MeCP2+ neurons increases, while the area of MeCP2- nuclei decreases. The slope of the MeCP2- soma area does not differ statistically from that of the MeCP2+ soma area [*F*_(1,44)_ = 3.41, *p* = 0.07; **Figure 10B**], however, the trend of diverging slopes is apparent.

To minimize inter-animal differences (i.e., differences associated with tissue preparation and fixation), MeCP2- areas were normalized to MeCP2+ areas in each slice and plotted across the proportion of MeCP2- neurons in the brain (**Figure 10C**). The normalized nuclear area of MeCP2- neurons was positively correlated to the proportion of MeCP2- neurons in the brain [*F*_(1,22)_ = 18.14, *p* = 0.0003]. The normalized soma area of MeCP2- neurons was also positively correlated to the proportion of MeCP2- neurons in the brain [*F*_(1,22)_ = 8.08, *p* = 0.0095]. These data suggest that animals with a highly skewed XCI ratio, favoring expression of the wild-type allele (<30% MeCP2-/>70% MeCP2+) have the most severe MeCP2- neuronal phenotype (17–22% smaller than MeCP2+). Animals with a balanced XCI ratio (∼50% MeCP2-/50% MeCP2+) have MeCP2- neurons with a comparatively less severe neuronal phenotype (11–17% smaller than MeCP2+).

### Soma and Nuclear Size of MeCP2 WT Versus MeCP2+ Neurons

**FIGURE 11:**
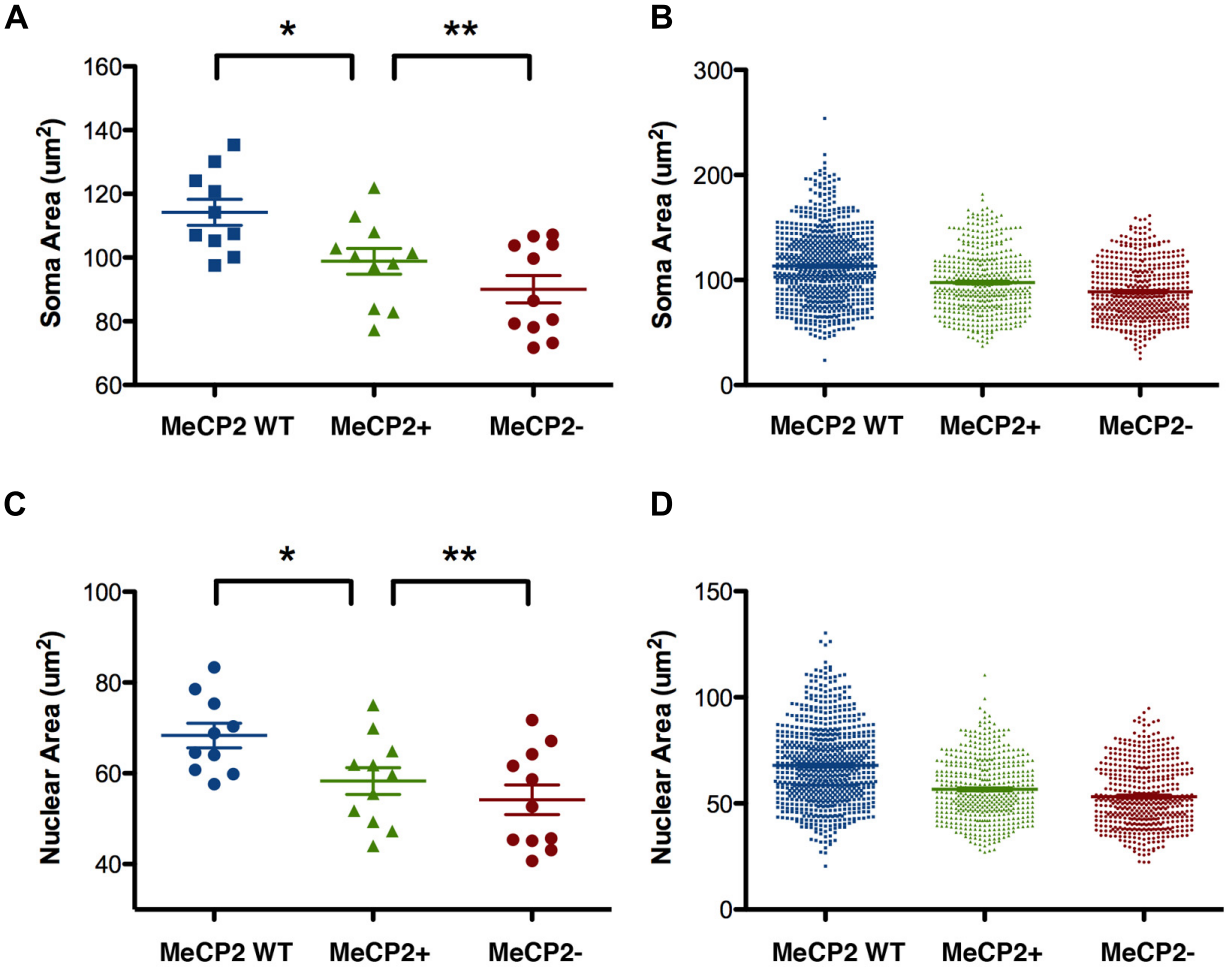
**Soma and nuclear size comparison between wild-type and heterozygous females. (A,C)** MeCP*2* WT Layer V neurons (wild-type) are larger than MeCP2+ neurons in heterozygous mice, which suggests a non-cell-autonomous effect of MeCP2 deficiency (MeCP*2* WT, *n* = 10 animals; MeCP2+, *n* = 11; MeCP2**-** *n* = 11). **(B,D)** MeCP2+ and MeCP2**-** neuronal populations in heterozygous brains each have a smaller proportion of large cells than the population of MeCP2 WT cells. The MeCP2**-** cell population also has fewer large cells than the MeCP2+ population in heterozygous brains. (total cells assessed MeCP2 WT, *n* = 761 neurons; MeCP2+, *n* = 482; MeCP2**-***n* = 502). *p < 0.05 and **p < 0.01.

For the study summarized in **Figures 7, 9**, and **10**, we did not have a comparison group of wild-type littermates, so it was not possible to test whether, MeCP2+ neurons were comparable to MeCP2 WT neurons, with respect to soma and nuclear size. Since a number of sources of error can potentially affect cell size, such as variation in cell shrinkage during fixation, we analyzed an entirely new group of mice comprising heterozygous females and their wild-type female littermates. As expected, comparison of MeCP2+ to MeCP2- soma and nuclear sizes in this new cohort of animals revealed a difference for the group mean data [**Figure 11**; mean soma difference 8.8 μm^2^, *p* < 0.0020, *n* = 11 animals; mean nuclear difference 4.1 μm^2^, *p* < 0.005). Within animal paired sample comparisons were effective to increase statistical significance and within animal soma and nuclear sizes were strongly correlated between genotypes (MeCP2+ regressed against MeCP2-, *R*^2^ = 0.97, *p* < 0.0001, nuclear *R*^2^ = 0.90 *p* < 0.0001), indicating that inter-animal variability contributes a substantial amount of variance to the group means.

MeCP2+ neurons in the heterozygous brain were smaller, on average, than MeCP2 WT neurons [mean soma difference 15.4 μm^2^, *p* < 0.032; nucleus 10.0 μm^2^, *p* < 0.038, *n* = 10 WT, *n* = 11 MeCP2+). Pairwise comparisons based on littermate status (siblings) or day of tissue preparation did not measurably improve the statistical discrimination of the difference between MeCP2 WT and MeCP2+ neurons. This confirms that the main source of variance in these experiments is intrinsic inter-animal variability in cell size, rather than variance in tissue preparation. When the overall cell size distribution is examined (**Figure 11B**), the tendency for the MeCP2- population to appear shifted toward smaller sizes when compared to the population of MeCP2+ cells, is once again apparent. Interestingly, the population of MeCP2+ cells also seems shifted toward small size when compared to the population of cells in *Mecp2*^+/+^ animals. Once again, the smallest cells in each population are similar in size.

## Discussion

### MeCP2- Neurons have Reduced total Dendritic Length and Fewer Third and Fourth Order Branches

**FIGURE 12:**
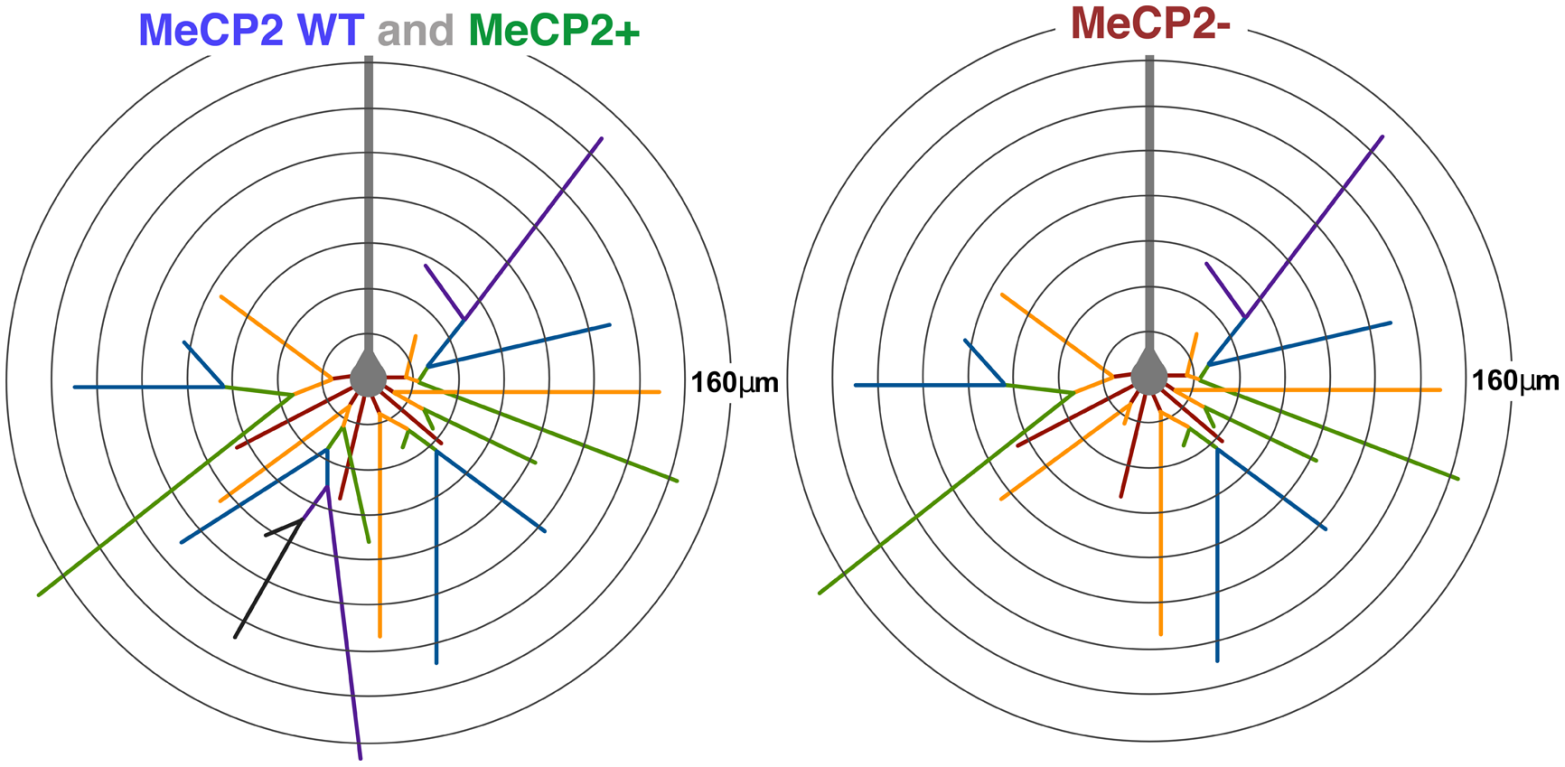
**Diagram of average MeCP2 WT, MeCP2+, and MeCP2**-** layer V pyramidal neurons.** The average MeCP2 WT and MeCP2+ neurons are equivalent across all morphological parameters investigated except soma and nuclear size. The average MeCP2**-** neuron (right) has approximately 15% less total dendritic length compared to either of the other genotypes. The average MeCP2**-** neuron has three fewer branch points, leading to a reduced number of higher order branches. The maximum branch order of MeCP2**-** neurons is also reduced. MeCP2- neurons have the same average maximum radial distance as both MeCP2 WT and MeCP2+ neurons. Somata of MeCP2**-** neurons are smaller than MeCP2+ especially in highly skewed XCI animals. Branch order: first = red, second = orange, third = green, fourth = blue, fifth = purple, sixth = black. Radii are 20 μm.

We find that MeCP2- layer V pyramidal neurons in heterozygous brains have shorter basal dendritic length, due to their having fewer third and fourth order branches compared to MeCP2+ neurons (see **Figure 12** for summary). This supports previous research in *Mecp2^tm1.1Jae^* male mice that also found a reduction in the number of higher order branches in layer V cells (Stuss et al., 2012). Within the basal dendritic compartment, the principal difference between neurons containing mutant MeCP2 protein compared to those expressing the wild-type allele in both *Mecp2^+/-^* and *Mecp2^+/+^* animals is a selective reduction in the number of third and fourth order branches. The similar pattern of reduced basal dendritic morphology of Layer V pyramidal neurons for both MeCP2- neurons in *Mecp2*^+/-^ females and male *Mecp2*^-^*^/y^* mice indicates that MeCP2 acts predominantly in a cell-autonomous manner to affect the dendritic morphology of this compartment of MeCP2- neurons. These results are consistent with other studies that found that callosal projection neurons from *Mecp2^-/y^* mice transplanted into either wild-type or *Mecp2^-/y^* brains had primarily cell autonomous reductions in dendritic morphology (Kishi and Macklis, 2009).

MeCP2+ neurons were indistinguishable from MeCP2 WT neurons in *Mecp2^+/+^* animals with respect to cumulative dendritic length, number of branch points, and maximum branch order versus distance from the soma. Therefore, we find that the basic neuronal morphology of Layer V basal dendrites is primarily affected by MeCP2 in a cell-autonomous manner. However, examination of some branch order analyses (**Figures 6B,D,E**) suggest MeCP2+ neurons have properties intermediate to those of MeCP2 WT and MeCP2-. Therefore, while cell-autonomous (genotype-specific) effects appear to dominate branch structure, additional non-cell-autonomous effects may also be influencing branching. An inherent difficulty with analysis of basal dendrite branching in layer V cortical neurons is the large variation in dendritic structure inherent to this neuronal population (Tsiola et al., 2003). A large number of neurons must therefore be sampled to obtain a representation of the “average” layer V pyramidal neuron making detailed comparisons between the dendritic arbors of neurons expressing wild-type MeCP2 in *Mecp2^+/+^* and *Mecp2^+/-^* animals a challenging task.

Our analysis of 35 heterozygous mice did not reveal a correlation between XCI ratios in the primary motor cortex and the severity of behavioral phenotype, although previous research has suggested that the severity of the RTT phenotype may be influenced by the proportion of Mecp2- neurons in the brain (Sirianni et al., 1998; Amir and Zoghbi, 2000; Plenge et al., 2002; Miltenberger-Miltenyi and Laccone, 2003; Young and Zoghbi, 2004; Weaving et al., 2005; Archer et al., 2007). We acknowledge that our phenotype severity was scored using gross behavioral tests, rather than more extensive testing which has been shown to reveal subtle abnormalities even in young female mice (Samaco et al., 2012), which do not ordinarily show obvious symptoms. Additionally, XCI ratios were determined using immunohistochemical analysis of MeCP2+ and MeCP2- neurons in the motor cortex of the brain, whereas other studies use blood or peripheral tissue to determine the XCI ratio, which has been reported to not necessarily correlate with the XCI in the brain (Gale et al., 1994; Sharp et al., 2000; Young and Zoghbi, 2004). Furthermore, although immunohistochemical determination of XCI may be more accurate, we limited our analysis to layer V pyramidal neurons, which appear to be particularly subject to wide fluctuations in the pattern of XCI inactivation (Wu et al., 2014).

### Soma and Nuclear Size of MeCP2- Neurons are Reduced in the Mecp2^+/-^ Brain

Analysis of soma and nuclear size revealed that the average MeCP2- soma/nucleus is smaller than that of the average MeCP2+ neuron, consistent with previous studies comparing male *Mecp2^-/y^* to *Mecp2^+/y^* mice (Kishi and Macklis, 2004; Fukuda et al., 2005). When the distribution of individual cell sizes from all animals are pooled, the MeCP2- population appears to primarily lack neurons comparable to the larger ones in the MeCP2+ population, while the prevalence of the smaller neurons in both genotypes is similar. This indicates that mutant MeCP2 may limit the size of neurons, but does not decrease the size of MeCP2- somata beyond that of the smallest subset of MeCP2+ neurons.

Recent studies have found that MeCP2 controls nuclear size in a largely cell-autonomous manner (Singleton et al., 2011; Yazdani et al., 2012). We find significant differences in the average sizes of somata and nuclei between MeCP2+ and MeCP2- neurons in the heterozygous brain, which appears to support this hypothesis. However, when comparing MeCP2+ cells in the heterozygous female brain to MeCP2 WT cells in the wild-type brain, we also find a significant difference in cell size, with neurons from the wild-type brain being considerably larger. This suggests additional non-cell autonomous mechanisms for the regulation of cell size by MeCP2, which may have a detrimental effect on MeCP2+ cell size in the heterozygous brain (Wu et al., 2014). This is an intriguing observation, since most studies to date have not examined neuron morphology in heterozygous females. Additionally, we found that while we needed only a small sample size to confidently avoid type 2 errors for comparisons between MeCP2+ and MeCP2- cells in heterozygous brains, a relatively large sample size (*n*>8) was required to demonstrate significant differences between MeCP2-positive cells in *Mecp2^+/-^ and Mecp2^+/+^* littermate pairs. This may partially explain why we were able to note differences in MeCP2+ cell size between wild-type and heterozygous animals, but were not able to note similar non-cell autonomous differences in dendrtitic morphology.

### An Effect of XCI Ratios on Soma and Nuclear Areas

Previous research has suggested that mutations in MeCP2 may act non-cell-autonomously to affect dendritic morphology through the secretion of inhibitory substances by MeCP2-deficient glia (Ballas et al., 2009; Maezawa et al., 2009; Maezawa and Jin, 2010). Braunschweig et al. (2004) report that as the number of MeCP2- neurons in the female *Mecp2^+/-^* brain increases, the expression of MeCP2 in MeCP2+ neurons decreases proportionally, suggesting that in addition to cell-autonomous genotype-specific effects, *Mecp2* mutations can negatively impact neurons through actions of surrounding MeCP2- neurons and/or glia. When comparing MeCP2 WT neuron size (soma and nuclear) in wild-type animals to that of MeCP2+ neurons in heterozygous females, (**Figure 11**) we see evidence of a non-cell-autonomous effect, in which the presence of MeCP2- cells in the brain is associated with reduced cell size for MeCP2+ neurons.

**FIGURE 13:**
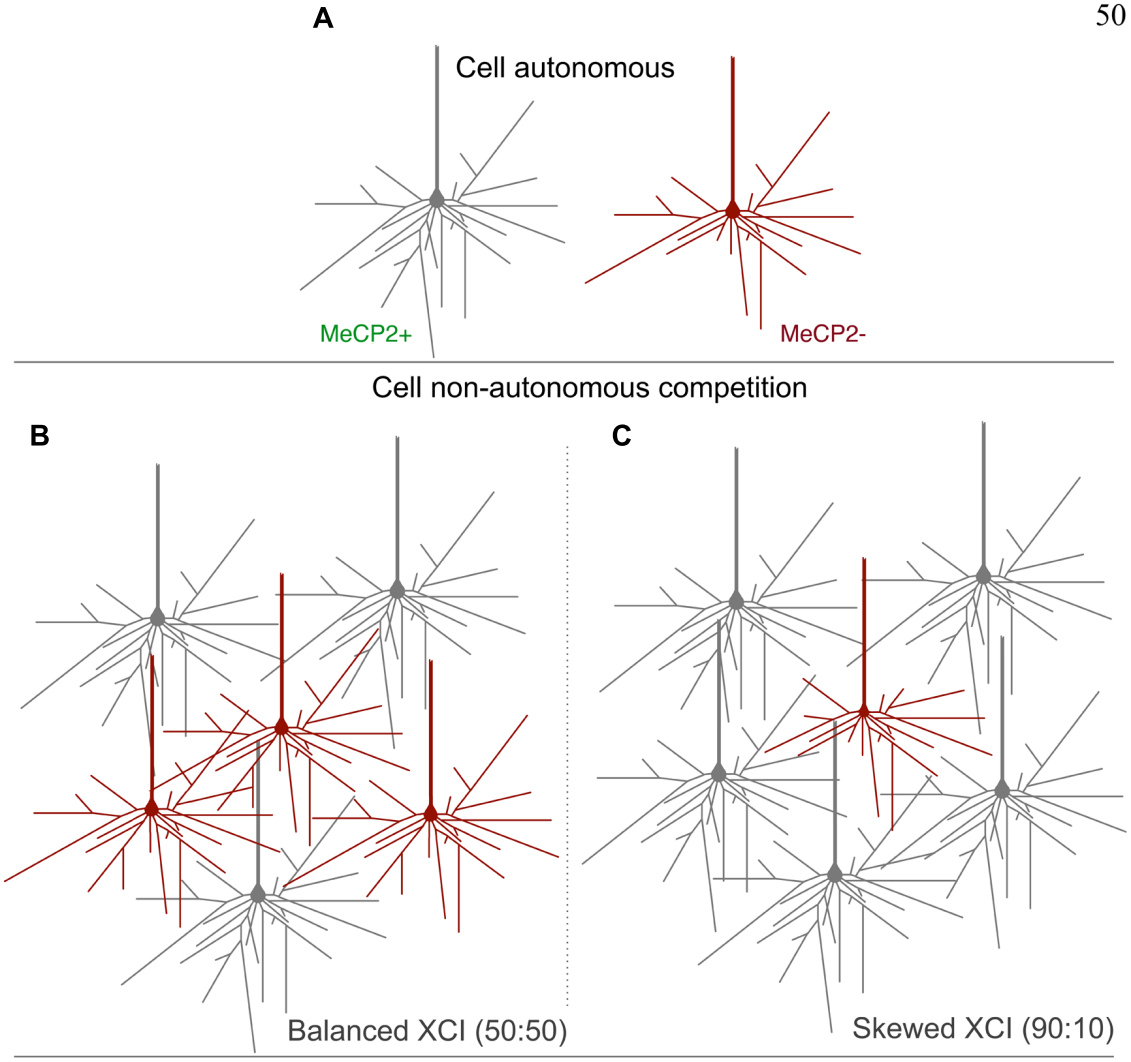
**Proposed model for cell non-autonomous effects of balanced and skewed XCI ratios. (A)** Cell- autonomous effects on the morphology of MeCP2- containing neurons, including reductions in dendritic length and fewer branch points compared to MeCP2+ cells, may render them less competitive for synaptic connections in the heterozygous brain. **(B)** A balanced XCI ratio may provide MeCP2- neurons more opportunity to make connections, as there are fewer large MeCP2+ neurons to compete with. This may reduce the severity of the morphological phenotype of MeCP2- cells. **(C)** A skewed XCI ratio favoring MeCP2+ neurons provides for a more competitive environment for synaptic connections. MeCP2- neurons are less able to compete and therefore have a more severe morphological phenotype.

Comparing MeCP2+ to MeCP2- neurons across XCI ratios presents a more complex picture, however, particularly when between-animal differences are normalized by within-animal comparisons. As the proportion of MeCP2- neurons in the brain decreases MeCP2+ neurons tend to get larger (consistent with non-cell-autonomous effects). However, MeCP2- soma and nuclear size tend to either not change or decrease, despite an increasing “wild-type” brain environment, resulting in a strong inverse relationship between XCI ratio and the ratio of MeCP2-/MeCP2+ cell size, shown in **Figure 10C**. Given that soma size has been reported to correlate with dendritic complexity (Kaufmann et al., 1995), these data may support a model in which MeCP2- neurons find it difficult to compete for connections in female brains with a highly skewed XCI ratio where the MeCP2+ neurons predominate. One hypothesis is that the consequent inability to compete for synaptic connections reduces the size of MeCP2- neurons to a greater degree than cell-autonomous effects alone. In contrast, in females with a balanced XCI ratio, the “Hebbian” synaptic environment is less competitive overall therefore allowing MeCP2- neurons to make more contacts with other neurons (**Figure 13**). Alternatively or additionally MeCP2- neurons may simply be unable to benefit from the more wild-type brain environment as the proportion of wild-type neurons increases with highly skewed XCI. Regardless of the cause, the result is that the cell-autonomous effects of MeCP2 mutation dominate even in a predominantly wild-type environment.

## Conflict of Interest Statement

The authors declare that the research was conducted in the absence of any commercial or financial relationships that could be construed as a potential conflict of interest.
